# Risky sexual practices and related factors among ART attendees in Addis Ababa Public Hospitals, Ethiopia: A cross-sectional study

**DOI:** 10.1186/1471-2458-11-422

**Published:** 2011-06-01

**Authors:** Yadeta Dessie, Mulusew Gerbaba, Abdo Bedru, Gail Davey

**Affiliations:** 1 Department of Public Health, College of Health and Medical Sciences, Haramaya University, Harar, Ethiopia; 2 Department of Population and Family Health, Faculty of Public Health and Medical Sciences, Jimma University, Jimma, Ethiopia; 3 Department of Epidemiology and Biostatistics, School of Public Health, Addis Ababa University, Addis Ababa, Ethiopia

## Abstract

**Background:**

Many HIV-positive persons avoid risky sexual practices after testing HIV sero-positive. However, a substantial number continue to engage in risky sexual practices that may further transmit the virus, put them at risk of contracting secondary sexually transmitted infections and lead to problems with drug resistance. Thus, this study was intended to assess risky sexual practices and related factors among HIV- positive ART attendees in public hospitals of Addis Ababa.

**Methods:**

A cross-sectional study was conducted among ART attendees from February to March, 2009. Questionnaire-based face-to-face interviews were used to gather data. SPSS software was used to perform descriptive and logistic regression analyses.

**Results:**

Six hundred and one ART attendees who fulfilled the inclusion criteria was included in the study and interviewed. More than one-third (36.9%) had a history of risky sexual practices in the three months prior to the study. The major reasons given for not using condoms were: partner's dislike of them, both partners being positive for HIV and the desire to have a child. Factors associated with risky sexual practices included: lack of discussion about condom use (Adjusted Odds Ratio (AOR = 7.23, 95% CI: 4.14, 12.63); lack of self-efficacy in using condoms (AOR = 3.29, 95% CI: 2.07, 5.23); lack of sexual pleasure when using a condom (AOR = 2.39, 95% CI: 1.52, 3.76); and multiple sexual partners (AOR = 2.67, 95% CI: 1.09, 6.57). Being with a negative sero-status partner (AOR = 0.33, 95% CI: 0.14, 0.80), or partners of unknown sero-status (AOR = 0.19, 95% CI: 0.09, 0.39) were associated with less risky practice.

**Conclusions:**

A considerable proportion (36.9%) of respondents engaged in unprotected sexual intercourse, potentially resulting in re-infection by a new virus strain, other sexually transmitted infections and onward transmission of the HIV virus. Health education and counseling which focuses on the identified factors has to be provided. The health education and counseling can be provided to these people at ART appointments on follow- up care. It can be provided in a one-on-one basis or through patient group educational discussions at the clinics.

## Background

Ethiopia is one of the sub-Saharan countries worst affected by the HIV/AIDS pandemic. According to the Ministry of Health of Ethiopia report published in 2008, approximately 1,345,970 people were living with HIV. In 2008, the national adult HIV prevalence was estimated to be 2.1% and a total of 34,936 people were newly infected with HIV, of whom 14,967 were adults [1]. The primary mode of transmission of HIV is through unprotected sex with infected individuals, which constitutes 88% of transmissions [2].

Public health experts emphasize the importance of addressing HIV prevention activities with HIV- infected persons while at the same time scaling up access to antiretroviral (ARV) treatment [3]. With increased access to highly active antiretroviral therapy (HAART), there has been a dramatic decline in morbidity and mortality from HIV disease [4]. Apart from their beneficial clinical effect, treatment advances may have unintended effects on sexual behavior [5]. Unprotected sexual intercourse among persons receiving HAART is a concern because of the risk of HIV-transmission to sero-discordant partners and the risk of re-infection with new drug resistant viral strains [6].

Many infected individuals avoid sexual practices likely to transmit HIV, but substantial numbers continue to engage in practices likely to transmit HIV [7], referred to in this article as "risky sexual practices". The concern is that, as more and more people with HIV live longer and healthier lives because of antiretroviral therapy, an increasing number of sexual transmissions of HIV may stem from those who know they are infected and still engage in unprotected sex [8]. Although individuals who derive therapeutic benefit from HAART may attain an improved quality of life and functional status, these gains may be accompanied by increases in risky sexual practices among individuals whose illness had previously inhibited those practices [9].

Meta analytic review of studies by Crepaz and his colleagues showed that between 10 and 60 percent of HIV-positive people continue to engage in unprotected sex [5]. Specifically 37% to 52% of HIV-positive women reported unprotected vaginal intercourse [10-12]. With regard to risky sexual practice and ART, mixed findings have been reported, some studies revealing a rise in risky practices once ART becomes available [13,14], and others no effect [15]. Yet others suggest that risky sexual practices may decrease among those on ART due to more intensive counseling [16]. Although there is clearly great variability in the findings of different studies, several suggest ongoing risky sexual practices among people with HIV [5,10-13]. Rerecent reports also indicate high incidence of sexually transmitted infections during ART treatment [13] and continued desire for fertility among those living with HIV [17,18], both suggesting the wish for and practice of unprotected sex.

Any individual's risk-taking behavior depends on the situation and how the individual assesses their risk [19]. Social dynamics, social interactions and the norms that govern interaction in a particular social context contribute to risky sexual practices [20]. Even when fully aware of the risks ahead, people make irrational choices because social interactions are power-based relationships [21,22]. An individual may fail to use a condom because a sexual partner refuses to do so and persuades them not to. Furthermore behaviors that are considered risky may be routine behaviors to some people [20,22]. Factors frequently reported to be associated with risky sexual practices included: partner related conditions [9,23-26]; psychosocial factors [23,27,28]; medical related factors; and behavioral correlates [29-31].

In Ethiopia in 2009, there were about 176,600 people on antiretroviral treatment [32]. As far as the authors are aware, little information exists on risky sexual practices among HIV-positive persons in Ethiopia. Deribe et al found that high risk sexual behaviors were common among HIV- positive people in Jimma [23], though this study was based in a single hospital. We planned this study to measure the prevalence of risky sexual practices and to identify factors related with them among people attending a range of public hospital ART clinics in Addis Ababa, Ethiopia.

## Methods

### Study setting

A cross-sectional quantitative study was conducted among people living with HIV attending ART clinics in public hospital in Addis Ababa from February to March 2009. According to the 2008 national report, the HIV prevalence in the city was 7.5% with a total of about 222,828 people infected with HIV [1]. There were 53 public and private ART-providing health institutions in Addis Ababa as per the November 2008 report, of which 9 were public hospitals, 23 were public health centers and the rest were public clinics. Overall, about 35,207 PLWHA were on ART at the time of the study [33].

### Participants

The study participants were ART attendees who had made two or more clinic visits, had tested positive at least three months ago, had been sexually active in the past three months and were 18 years of age or above. Patients who were mentally ill, unable to communicate, seriously ill, or did not fulfill the inclusion criteria were excluded from the study.

Seven public hospitals were included in the study. They included Black Lion Hospital (2,352 registered at the ART clinic), Minilik II Hospital (1,592), Ras Desta Hospital (851), St Paulos Hospital (2,680), St. Peter Hospital (1,520), Yekatit 12 Hospital (1,955), and Zeweditu Hospital (5,320). Two hospitals were not included; one because it only provided Prevention of Maternal to Child Transmission (PMTCT) services and the other one due to administrative process challenge in getting permission to conduct the study.

The sample size was calculated using the single population formula by assuming prevalence of unprotected sex (p) to be 50%, the marginal error to be 4%( d), and 95% Confidence Interval (CI) giving a sample size of 601. The sample from each hospital was allocated proportionally to the number of clients on ART at each institution. Every client who came for ART services, fulfilled the inclusion criteria and consented to participate was interviewed till the required sample size was met. In the study period, 1,781 patients were consecutively approached to determine eligibility, of whom 601 fulfilled the inclusion criteria and consented to participate. The reasons for non-eligibility were: not sexually active within past 3 months (1120); less than 18 years old (42) and did not agree to participate (18).

### Data Collection

A structured questionnaire containing different components derived from a range of behavioral constructs and studies [5,21,34-37] was used. The questionnaire was first prepared in English, then translated into the national language, Amharic, and then translated back to English to check for consistency and phrasing of difficult concepts. Trained ART adherence counselors and nurse counselors were used to conduct the interviews and collect the data. The data collectors and supervisors were trained in the methods, objectives, and other technical aspects of the study before data collection commenced. Pre-testing was conducted with 31patients (5% of anticipated sample), who were not included in the main study. Questions causing difficulty in the pre-test were rephrased and corrected.

### Measurements

The main outcome variable for the study was "risky sexual practices": condom-unprotected sex with either HIV-negative, positive or unknown sero-status partners in the previous three months. The independent variables were socio-demographic characteristics including age, sex, ethnicity, education, religion, marital status, occupation and income status; relationship factors including the number of sexual partners, types of sexual partner and any discussion about condom use, partner's HIV sero-status and their disclosure status. Other independent variables included medically related factors like safer sex beliefs and the duration of HIV diagnosis and start of ART, safe sex beliefs about ART and safer sex knowledge, pleasure and effectiveness. Psycho-social factors included stigma, active substance and alcohol use; behavioral factors included self-efficacy to use a condom, and general social support by family and friends.

*Condom use self-efficacy *was assessed using five items derived from a scale developed by Brafford and Beck [34] address the capacity of individuals to make their partner use condoms, their ability to wear and remove condoms, and had responses of 1 (strongly agree) to 5 (strongly disagree), and 3 as "don't know". A score below the mean total sum value of the respondents was taken to mean good self-efficacy to use a condom.

*General social support was *assessed on a five item scale derived from that developed by Cutrona and colleagues [35]. It addresses the perceived social support that an individual gets from others with response options of 1 (strongly disagree) to 4 (strongly agree). A score above the mean total sum was taken to represent high perceived general social support.

Safer sex knowledge, safer sex effectiveness and safer sex pleasure during condom use

Safer sex knowledge was assessed using a four- item scale (knowledge related to re-infection with other strains of the virus and STIs), while safer sex effectiveness was assessed using a three- item scale on how effective condoms were in preventing HIV and STIs). Safer sex pleasure during condom use used a three item-scale focusing on how condoms changed sexual pleasure.

These scales were adapted from those used in a study of correlates of unprotected sex among HIV positive people [36] with response options from 1 (strongly agree) to 5 (strongly disagree) and 3 (don't know). Since the safer sex knowledge scale showed a skewed distribution, the median was taken and values above the median score were taken as a lack of safer sex knowledge. For the safer sex effectiveness scale and safer sex pleasure scale, scores above the mean were taken as negative safer sex effectiveness and safer sex pleasure.

*Stigma *was assessed in two ways; the first was enacted stigma assessed with nine Yes (coded as 1)/No (coded as 0) response option questions addressing stigma encountered after testing positive. The second was perceived stigma with seven Yes (coded as 1) and No (coded as 0) questions related to avoidance, social rejection, and shame three months prior to the study adapted from scale used in one study [37]. In both cases, scores above the mean indicated enacted and perceived stigma.

In this study **'**risky sexual practice' was defined as condom-unprotected sex with either HIV-negative, positive or unknown sero-status partners in the previous three months; a ' steady partner' was one with whom the respondent had a regular sexual relationship and who was perceived by the respondent to be the spouse or regular boy/girl friend for more than three months; while a 'casual partner' was one other than the regular partner with whom the respondent had sexual intercourse in the past three months with or without payment.

## Data analysis

The data were entered and analyzed using SPSS version 16. Descriptive statistics were used to present frequency distributions. The Chi-squared test was used to evaluate the association between current condom use levels and levels of condom use before testing positive. Bivariate analysis was employed to identify factors associated with risky sexual practices. Multiple logistic regression analysis was performed for those factors that showed a statistically significant association in bivariate analysis and investigate independent predictors by controlling for possible confounders.

## Ethical considerations

Ethical clearance was obtained from the Addis Ababa University Institutional Review Board (IRB). Respondents were informed of the purposes, procedures, risks and benefits, and the private and confidential nature of the study. Participation was voluntary and declining participation would not bring any adverse consequences in terms of service provision at the ART clinic. Written informed consent was obtained from each respondent.

## Results

### Socio demographic characteristics

The total sample included 601 HIV positive ART attendees who fulfilled the inclusion criteria and agreed to participate. More than half 331 (55.1%) were females. The mean age of the respondents was 33.4 ± 6.5 (SD) years, and a large percentage of the respondents (57.7%), were aged 26-35 years. By ethnicity, 264 (43.9%) were Amhara, and 163 (27.1%) Oromo. More than half, 324 (53.9%), had attended school grades 7-12. These people were less likely to have engaged in unprotected sex (p = 0.03) than those with less schooling. Most, 419 (69.7%), were Orthodox Christian followers, 98 (16.3%) Muslims and the remainder were Protestants and Catholics. With regards to their marital status, 384 (63.9%) were married, 142 (23.6%) had never married and the rest were divorced, separated or widowed. Marital status did not show a statistically significant association with risky sexual practices. Over half, 331 (55.1%), were unemployed and for 180 (30.0%) respondents, the average monthly income was below ETB 500 ($42 in 2009). For more detail see Table 1.

**Table 1:** Socio-demographic characteristics of respondents on ART in Addis Ababa public hospitals, 2009

Characteristics	Frequency N (%)	Unprotected Sex (n/N)	OR (95% CI)	P-value
**Sex of the respondents**				
Male	270 (44.9)	102/270	1.0	
Female	331 (55.0)	120/331	0.94 (0.67,1.31	0.70
**Age (years)**				
≤25	48(8.1)	23/48	1.00	
26-35	348(57.7)	116/348	0.54(0.29,0.99)*	0.50
≥36	205(34.1)	83/205	0.74(0.39,1.39)	0.35
**Education status**				
≤6 grade	152 (25.3)	66/152	1.00	
7-12 grade	324 (53.9)	108/324	0.65 (0.41,0.97)	0.034
>12 grade	125 (20.8)	48/125	0.81 (0.50,1.32)	0.398
**Marital status**				
Married	384 (63.9)	145/384	1.00	
Unmarried	142 (23.6)	53/142	0.98 (0.66,1.46)	0.93
Divorced	30 (5.0)	11/30	0.95 (0.44,2.06)	0.91
Separated	14 (2.3)	3/14	0.45 (0.12,1.64)	0.23
Widowed	31 (5.2)	10/31	0.79 (0.36,1.71)	0.54
**Employment status**				
Employed	270 (44.9)	99/270	1.00	
Non employed	331 (55.1)	123/331	1.02 (0.73,1.43)	0.90
**Income status**(Eth. Birr)*				
Have no income	132 (30.0)	42/132	0.87 (0.50,1.51)	0.61
Not specified	90 (15.8)	42/90	1.57 (0.87,2.83)	0.13
≤500	180 (15.0)	66/180	1.04 (0.62,1.74)	0.89
501-999	104 (17.2)	37/104 34/95	0.99 (0.55,1.77)	0.98
≥1000	95 (24.9)		1.000	

*12 Ethiopian Birr = 1 $ USD in 2009, statistically significant at (p < 0.05), OR, Crudes Odds Ratio

### Sexual practices and partner's related characteristics

In the three months prior to the study, nearly two-thirds (63.1%) of respondents had used condoms in a consistent manner while 91 (15.1%) had used them inconsistently and 131 (21.8%) had never used a condom (Table 2). More than one-third (36.9%) had one or more sexual encounter(s) without using a condom, of which 77.0% were with a steady partner, 16.8% with a casual partner and 6.3% with steady and/or casual partners. Those experiencing mixed partners were more likely to engage in unprotected sexual intercourse than those with either a steady or a casual partner (p = 0.002).

**Table 2:** Patterns of condom use three months prior to the study among respondents ART in the public hospitals of Addis Ababa, 2009

Condom use pattern 3 months prior to study	Frequency	**Condom use before testing HIV**^**+**^			
	N (%)	Irregular users	Non- Users	**X**^**2**^	P-Value
Consistent user(always used)	379 (63.1%)	123 (32.5% )	156 (67.5% )		
Inconsistent user(irregularly used)	91 (15.1%)	37 (40.7%)	54 (59.3%)	9.95	0.007 *
Non- users (not used at all)	131 (21.8%)	28(21.4%)	103 (78.6%)		

*statistically significant (p < 0.05)

Ninety percent of the respondents reported a single partner while the rest reported multiple partners. Multiple partners, defined as two or more partners, was significantly associated with unprotected sex (p = 0.001). Of those who reported a single partnership, 488 (90.2%) had a steady partner, whereas 53 (10.8%) had a casual partner. Among those with multiple partners, the majority (60%) were casual partners, while 19 (32.67%) reported both steady and casual partners, these latter being more likely to engage in unprotected sex (p = 0.002). With regards to their partner's sero-status, 492(81.9%) were aware of their partner's serostatus 430 (87.5%) sero-positive and 62 (12.5%) sero-negative). The rest 105(17.5%) did not know their partner's sero-status and 4 (0.7%) reported both positive and unknown sero-status. Those with a sero-negative partner were less likely to engage in unprotected sexual intercourse (p < 0.001). Those who engaged in little or no discussion with their partner about safe sex were more likely to engage in unprotected sex (24% vs 75%) and the difference was statistically significant at (p < 0.001), see Table 3.

**Table 3:** Sexual partner relationship characteristics among respondents on ART in the public hospitals of Addis Ababa, 2009

Characteristics	Frequency (%)	Unprotected sex (n/N)	OR (95% CI)	P-Value
**Number Current partner(s)**				
Single	541 (90.0)	188/541	1.0	
Multiple	60 (10.0)	34/60	2.47(1.43,4.22) *	0.001
**Life partner(s) before testing positive**				
One	294 (48.8)	101/294	1.00	
Two	162 (27.0)	55/162	0.98(0.62,1.47)	0.90
Three and above	146 (24.5)	66/146	1.57(1.04,2.35) *	0.03
**Condom use before testing positive**				
Yes	188 (31.3)	65/188	1.00	
No	413 (69.7)	157/413	1.16(0.81,1.66)	0.42
**Types of current partner(s)**				
Steady partner	493 (82.1)	171/494	1.00	
Casual partner	89 (14.8)	37/89	1.33 (0.84,2.12)	0.213
Both	19 ( 3.1)	14/19	5.27(1.87,14.89) *	0.002
**Discussion about safe sex with partner**				
Yes	448 (74.5)	107/448	1.00	
No	153 ( 25.5)	115/153	9.65(6.30,14.77) *	<0.001
**Disclosure to sex partner(s)**				
Yes	496 (82.5)	177/496	1.0	
No	105 (17.7)	45/105	1.35(0.88,2.07)	0.17
**Partner's HIV Sero-status**				
Positive	430 (77.1)	175/430	1.00	
Negative	62 (11.1)	9/62	0.25(0.12,0.56) *	<0.001
Unknown	105 ( 17.5)	35/105	0.73(0.47,1.14)	0.17

Statistically significant at (p < 0.05), OR, Crudes Odds Ratio

### Reported reasons for not using condoms

With multiple responses possible, reasons stated for not using a condom included - partner did not want to use (25.1%); my partner was also HIV+ (24.3%); desire to have a child (18.1%); sex did not feel the same with a condom (12.5%); not aware of the importance of condoms after sero-conversion (9.7%); were drunk and didn't remember to use a condom (5.7%); had no condom available (3.4% ); use was against their religion (3.4% ); fear of asking partner (2.8%); and thoughts that the partner did not have an STI (1.1%). Less common responses included not wanting to use, partner had started ART treatment and the use of another method of family planning.

### Medical, psycho social and behavioral characteristics

The majority of the study subjects (64.8%) had survived over 24 months since testing positive, 18.3% had survived 13-24 months, and the rest had survived 12 months or less. As far as ART medication was concerned, 38.9% had started less than 12 months before the study, 37.1% more than 24 months, and the rest were between 12 months and 24 months. Time since testing positive and duration on ART did not show any significant association with risky sexual practices. Respondents were asked whether there 'was less concern to practice safe sex because of ART treatment' and about one fourth (23%) agreed with this; those agreeing were more likely to have had unprotected sex (p < 0.001).

The majority (62.4%) of participants felt they had self-efficacy in condom use; those without condom use self efficacy were more likely to have engaged in unprotected sex (21.1% v 63.3%, p < 0.001). A total of 388 (64.6%) of the respondents perceived general social support. One third had experienced enacted stigma since they had tested positive and nearly half (49.1%) had perceived stigma in the three months prior to the study. Those who had experienced enacted stigma and perceived stigma were more likely to have engaged in unprotected sex (p = 0.02 and p = 0.01) respectively than those who had not, though not reach significance after adjusting for possible confounders.

One fifth of the respondents had a history of alcohol use in the last three months. The majority (68.9%) used alcohol two or fewer times per week and the rest three or more times. Those who consumed alcohol were more likely to have engaged in risky sexual practice (p < 0.001). Forty-five (7.2%) had a history of substance use, of whom 40 used *khat *(though the majority (72.5%) of these used it less than twice per week); 27 used cigarettes (44.5% used three or more times per week); 6 reported using *hashish' *(cannabis) and 9 used *shisha *(a less active narcotic smoked (sucked) through a tube like apparatus). Respondents who had used these substances were more likely to have engaged in unprotected sex in the bivariate analysis (p = 0.004, Table 4), though the association did not reach significance after adjustment.

**Table 4:** Medical, psychosocial and behavioral characteristics among respondents on ART in public hospitals of Addis Ababa, 2009

Characteristics	Frequency (%)	Unprotected sex (n/N)	OR (95% CI)	P-Value
**Time since testing positive(months)**				
3-12 months	101 (16.8)	33/101	1.0	
13-24 months	110 (18.3)	42/110	1.27 (0.72,2.24)	0.40
>24 months	389 (64.7)	146/389	1.24 (0.78,1.97)	0.37
**Duration since start of ART(months)**				
<12 months	234 (38.9)	90/234	1.0	
13-24 months	144 (23.9)	53/144	0.93 (0.61,1.43)	0.75
>24 months	223 (37.1)	79/223	0.88 (0.60,1.28)	0.51
**Reduced need to practice safe sex because of ART**				
Agree	138 (23.0)	45/138	2.10 (1.43,3.09) *	
Disagree	462 (77.0)	174/462	1.0	<0.001
**Negative safer sex knowledge**				
No	414 (68.9)	123/414	1.0	
Yes	187 (31.1)	99/187	2.66 (1.86,3.80)*	<0.001
**Negative safer sex effectiveness**				
No	431 (71.7)	137/431	1.0	
Yes	170 (28.3)	85/170	2.15 (1.49,3.08)*	<0.001
**Negative safer sex pleasure**				
No	334 (55.6)	80/334	1.0	
Yes	137 (44.4)	142/137	3.61 (2.55,5.11) *	<0.001
**Self-efficacy to use condom**				
Yes	375 (62.4)	79/375	1.00	
No	226 (37.6)	143/226	6.46 (4.47,9.32)*	<0.001
**General social support**				
Yes	388 (64.6)	133/388	1.00	
No	213 (35.4)	89/213	1.38 (0.98,1.94)*	0.69
**Enacted stigma**				
Yes	180 (29.9)	79/180	1.52 (1.06,2.17)*	
No	421 (70.1)	143/421	1.0	0.021
**Perceived stigma**				
Yes	195 (32.4)	125/195	1.58 (1.12,2.21)*	0.007
No	316 (67.6)	97/316	1.0	
**Alcohol use in the last three months**				
Yes	140 (23.3)	65/140	2.0 (1.35,2.97) *	<0.001
No	461 (77.7)	157/461	1.0	
**Other substance use **^**a**^				
No	556 (92.7)	199/556	1.00	
Yes	45 (7.3)	23/45	1.88 (1.02,2.45)*	0.004

*Statistically significant at (p < 0.05), OR, Crudes Odds Ratio ^a^Substances were khat, Cigarettes, Shisha and hashis

### Multiple logistic regressions of factors related to risky sexual practice

In order to identify independent predictors, variables that showed statistical significance in bivariate analyses were considered for multiple logistic regressions. Before the multiple logistic regressions, multicolinarity was checked with Pearson Correlation. In the case correlation values above (0.7) obtained, one of the variables was discarded. All factors except number of sexual partners and type of current partner (which were highly correlated) were simultaneously entered into multiple logistic regressions. Age, educational status, life partner before testing positive, concern of reduced need to use condom because of ART, safer sex effectiveness and knowledge, both enacted stigma and perceived stigma, alcohol and other substance use were no longer significant after controlling for possible confounders. Those reporting multiple partners (AOR = 2.67, 95%CI: 1.09, 6.57), those who did not discuss condom use or safe sex (AOR = 7.23, 95% CI: 4.14, 12.63), having negative safer sex pleasure (AOR = 2.39, 95% CI: 1.52, 3.76), and those lacking condom use self-efficacy (AOR = 3.29, 95% CI: 2.07, 5.23) were more likely to engage in unprotected sex. On the other hand, those who had a sero-negative partner (AOR = 0.33, 95% CI: 0.14, 0.80) or a partner of unknown sero-status (AOR = 0.19, CI 95% CI: 0.09, 0.39), were less likely to practice unprotected sex (Table 5).

**Table 5:** Condenced multiple logistic regression of an exploratory variable of the risky sexual practice among respondents on ART in Addis Ababa Public Hospitals, 2009

Characteristics	Condom unprotected sex					
	No (%)	Yes (%)	COR(95% CI)	P-Value	AOR(95% CI)	P-Value
**Number of Current partner(s)**						
Single	353 (62.5)	188 (34.8)	1.00		1.00	
Multiple	26 (43.3)	34 (56.7)	2.46 (1.43,4.22)*	0.001	2.67 (1.09,6.57)*	0.032
**Discussion about safe sex**						
Yes	341 (76.1)	107 (23.9)	**1.00**		**1.00**	
No	38 (24.8)	115 (75.2)	9.65(6.30,14.77)*	<0.001	7.23(4.14,12.63)*	<0.001
**Partner's HIV status**						
Positive	255 (59.3)	175 (40.7)	**1.00**		**1.00**	
Negative	53 (85.5)	9 (14.5)	0.25 (0.12,0.56)*	<0.001	0.33(0.14,0.80)*	0.014
Unknown	70 (66.7)	35 (33.3)	**0.73 (0.47,1.14)**	0.17	0.19(0.09,0.39)*	0.001
**Negative safer sex pleasure**						
No	254 (66.0)	80 (24.0)	**1.00**		**1.00**	
Yes	125 (46.8)	142 (53.2)	3.61 (2.55,5.11)*	<0.001	2.39 (1.52, 3.76)*	<0.001
**Self-efficacy to use condom**						
Yes	296 (78.9)	79 (21.1)	**1.00**		**1.00**	
No	83 (36.7)	143 (63.3)	6.46 (4.47,9.32)*	<0.001	3.29 (2.07, 5.23)*	<0.001

**Adjusted for socio-demographic, partner related, medical and safer sex beliefs, psychosocial and behavioral characteristics. *statistically significant (p < 0.05). COR: Crude Odds Ratio, AOR: Adjusted Odds Ratio,

## Discussion

Many people who learn they are HIV infected alter their behavior to reduce their risk of transmitting the virus [38]. Among those who remain sexually active, many complain of having trouble in using a condom regularly and there are still men and women living with HIV who experience difficulty maintaining safe sex even though the rates vary widely according to the specific group, the period under observation and the definition of safe sex [6,39]. This study revealed that 36.9% of the respondents had condom-unprotected ('risky') sexual intercourse within the three months prior to the study. This is higher than study reports from South Africa and the United States, where the prevalence of risky sex was 30% and 23% respectively [40,41]. The reasons might be related to the high reported intention to have a child in this study, and the fact that most of the respondents were in a marital relationship, unlike the other studies. A steady partnership was the commonest partner type reported (by 82% of participants), a proportion similar to that in a study in Botswana [25]. The 10% who reported multiple partners in this study was higher than the 5.6% in a study from South Africa [40] and these differences are possibly attributable to the study setting.

Condom-unprotected sexual intercourse occurred most often (79.3%) between sero-positive partners. The likelihood of risky sexual practice was lower among those who knew their partner's status to be HIV negative or did not know it, similar to findings reported from the United States [26], but in marked contrast to findings from South Africa, the Dominican Republic, and Jimma, Ethiopia, where condom-unprotected encounters were more likely to occur among those with unknown sero-status partners [23,26,36,42]. At this juncture, it should be critically noted that 4.1% and 16.2% of the unprotected encounters were with partners perceived to be sero-negative or of unknown sero-status, respectively, which might contribute to new infection or re-infection with a new strain of HIV. Unlike the Botswana study [25], where no significant difference was detected with respect to the number of partners, here multiple partners were about two times more likely to have condom-unprotected sex. Like the studies from South African and Botswana [26,40], there were no significant differences in risky sex by gender, marital status, employment status or income level.

The most important aspect of interpersonal communications related to prevention of HIV transmission is whether sexual partners have explicitly discussed the issue of safer sex and have reached agreement about it [43]. In this study, those who reported discussing condom use (safe sex) with their sex partner used condoms more consistently than their counterparts.

The reasons primarily reported in studies conducted in the Dominican Republic and Jimma, Ethiopia [23,24] for not using condoms were also identified in the current study. About twenty five percent reported their partner did not want to use a condom, and about twenty four percent said that their partner being sero-positive was the reason for not using condoms. One important finding here is that for fifty- two (18.1%) respondents, the main reason for not using a condom was their or their partner's desire for a child. In line with another study [43] since diagnosis and duration on ART was not found to be significantly associated with unprotected sex.

The issue that condoms take away sexual pleasure was illustrated in this study: those with negative safer sex pleasure were less likely to be consistent users of condoms, which is in line with findings from previous studies [28,36] that negative sexual pleasure in using condoms was significantly associated with unprotected sex. Many of the factors predicting unprotected sex in the general population also holds true for seropositive people. In this regard, those who lacked self-efficacy in condom use were about three times as likely to have condom unprotected sex than those who had good (high) self-efficacy. Unlike some other studies in which perceived stigma was associated with unprotected sex [23,28], in this study, stigma failed to attain significance after controlling for the role of possible confounders.

## Conclusions

Risky sexual practices defined as condom unprotected sexual intercourse is highly prevalent among this group of Ethiopian HAART users. More than one third of respondents had risky sex in the three months prior to the study and the main reasons given were partners' dislike, both partners being HIV infected and the desire for a child. Those who had little discussion about safe sex, negative safe sex pleasure, low self-efficacy to use condoms and multiple partnerships were more likely to have unprotected sex. There is a worry that condom-use fatigue may be occurring simultaneously with prolonged life through the use of ART. Behavioral change health education and counseling adapted to the specific needs of each patient must be programmed. Interventions must encourage free and explicit discussion among partners about safe sex and enhance positive attitudes toward condom use. Health education and counseling might be provided to these people at ART appointments and in follow- up care. This might either be provided on a one-on-one basis or through patient group discussions

The study has limitations. First, the consecutive approach used in recruiting participants may limit the external generalizablity of the study. Second, the cross-sectional study design makes it difficult to determine the direction of causality, and a prospective design is recommended to confirm the factors identified. Thirdly, the sensitive nature of sexuality may result in social desirability bias, likely to lead to underestimate of the prevalence of risky sexual practices. Lastly, not including private hospitals may have led to selection bias and again, may limit generalizability.

## Competing interests

The authors declare that they have no competing interests.

## Authors' contributions

YD participated in the design of the study, performed the data collection, performed the statistical analysis and served as the lead author of the manuscript. GD participated in the design of the study and contributed to drafting the manuscript. AB participated in the design of the study and helped perform the statistical analysis. MG participated in drafting and preparing the manuscript. All authors read and approved the final manuscript.

## Pre-publication history

The pre-publication history for this paper can be accessed here:

http://www.biomedcentral.com/1471-2458/11/422/prepub
